# In vitro muscle contraction: A technical review on electrical pulse stimulation in C2C12 cells

**DOI:** 10.1113/EP092677

**Published:** 2025-08-08

**Authors:** Mark R. C. van de Meene, Anita M. van den Hoek, Roeland Hanemaaijer, Lars Verschuren, Jelle C. B. C. de Jong

**Affiliations:** ^1^ Department of Microbiology and Systems Biology The Netherlands Organization for Applied Scientific Research (TNO) Leiden The Netherlands; ^2^ Department of Metabolic Health Research The Netherlands Organization for Applied Scientific Research (TNO) Leiden The Netherlands

**Keywords:** contraction, electrostimulation, EPS settings, exercise, in vitro model, muscle, myotubes, translational

## Abstract

Electrical pulse stimulation (EPS) of skeletal muscle cells is increasingly used to model exercise In vitro. The murine C2C12 myotube system has become a common platform for such studies, yet wide variability in EPS protocols hampers reproducibility and cross‐study comparisons. In this technical review, we analysed 54 peer‐reviewed studies that employed EPS in C2C12 and extracted used EPS protocols to provide an overview of the most commonly used settings for the EPS parameters (pulse duration, frequency, voltage and stimulation duration). Additionally, we summarized the biological processes investigated in these studies to illustrate the range of research topics typically addressed using this model. The majority of studies used 2 ms pulses at 1 Hz and moderate voltages (10–20 V), often over 24 h of stimulation. Glucose uptake was the most commonly assessed endpoint, followed by AMPK activation, inflammation and mitochondrial adaptations. Correlation analyses revealed interdependence between pulse duration, voltage and EPS duration, indicating that these parameters are often balanced to avoid excessive or suboptimal stimulation. While frequency was largely standardized, voltage and pulse duration showed greater variation. Our findings underscore the need for more detailed parameter reporting and deliberate protocol design aligned with specific experimental objectives, such as mimicking endurance‐ or resistance‐type exercise stimuli. This review serves as a resource for selecting EPS parameters tailored to specific biological processes and encourages standardization to improve translational relevance.

## Abstract (highlights)

- **What is the central question of this study?**
  In electrical pulse stimulation (EPS) protocols in C2C12 myotubes, What are the commonly used parameters, experimental approaches, and reported biological outcomes in C2C12 myotube electrical pulse stimulation (EPS) protocols aimed at modeling skeletal muscle contraction and exercise physiology?
- **What is the main finding and its importance?**
  Analysis of 54 studies revealed common EPS settings (2 ms pulse, 1 Hz, 20 V, 24 h). A higher voltage was associated with a longer length of stimulation, but a lower total duration of EPS. Protocols varied depending on the purpose of the research. This review offers a valuable guide for protocol selection and promotes standardization across future EPS studies.

## 1 INTRODUCTION

Regular physical activity plays a crucial role in promoting overall health and preventing or even reversing many chronic diseases including musculoskeletal, cardiometabolic, neurological and even psychiatric disorders (Anderson & Durstine, 2019; Duclos, 2021; Schuch & Vancampfort, 2021). Consequently, exercise reduces mortality and morbidity, improving both lifespan and quality of life; which is increasingly important due to our ageing society (Feng et al., 2023; Hegde, 2018). Given exercise's health benefits, a better understanding of the underlying mechanisms that mediate these beneficial effects is important and could aid in developing pharmacological or nutritional therapies that mimic or enhance the effects of exercise interventions. However, these mechanisms remain incompletely understood, and translational In vitro exercise models could help to unravel the underlying pathways.

During recent decades, the murine myoblast cell line C2C12 has consistently demonstrated its value as an In vitro muscle model. The C2C12 cells can In vitro be differentiated into myotubes which can contract upon receiving electrical pulse stimulation (EPS) (Nedachi et al., 2008). EPS improves the translational value of the In vitro system by mimicking physiological muscle activity more accurately than a static In vitro muscle model (Evers‐Van Gogh et al., 2015; Lee et al., 2022). Indeed, the C2C12‐EPS model has previously been successfully combined with in vivo studies both in mice and in humans to validate the translatability of results obtained In vitro. For instance, Wu et al. (2023) found that liver transthyretin (TTR) disrupts myotube composition and physiology in C2C12 cells through mechanisms which were subsequently reversed in *TTR^−/−^* mice. Similarly, Lautaoja‐Kivipelto et al. (2024) managed to confirm that upregulation of certain micro‐RNAs as previously found in in vivo studies also occurred in their C2C12‐EPS model. Moreover, a human trial involving diabetic patients found that a specific *NDUFB6* single nucleotide polymorphism (SNP) (rs540467) conferred poorer response to an exercise intervention than non‐carriers. When this group introduced the SNP into a C2C12‐EPS model, they observed reduced mitochondrial function thereby potentially explaining partly the poorer response to physical activity in these diabetic patients (Pesta et al., 2021). Thus, these results suggest that the C2C12‐EPS model provides mechanistic information which is translatable to humans.

Nonetheless, a pertaining issue with exercise‐like EPS models is the large variety of different EPS parameters and protocols that are applied in research. This diverse range of protocols arises partly due to the fact that studies aim to mimic different types of exercise that exert varying physiological stimuli, such as resistance training compared to endurance exercise (Carter & Solomon, 2019). Consequently, EPS‐mediated effects exhibit significant discrepancies, limiting the comparability of results from different studies. Moreover, determining the suitable EPS parameters in future research remains challenging due to the lack of standardization in protocol design. Therefore, we aim to review the EPS protocols described in current literature using the C2C12 cell line and evaluate the corresponding study outcomes for each protocol. Through this approach, we seek to evaluate EPS protocols currently being used and to identify the common and uncommon EPS protocols. We also aim to outline the biological parameters studied using EPS and the experimental techniques used to measure the respective biological parameters.

## 2 METHODS

### 2.1 Literature search strategy and data extraction

A literature search was performed in Embase (Scopus) on 7 February 2025 using the following search string: (TITLE‐ABS‐KEY(‘EPS’ OR ‘electrical stimulation’ OR ‘electrical pulse stimulation’))AND(TITLE‐ABS‐KEY(‘C2C12’)). The search was limited to English‐language articles and included studies published up to the date of the search. Only original research articles reporting original experimental data were included in which EPS was used specifically on C2C12 cells. In addition to the database results, backward citation tracking was performed on the reference lists of relevant articles to identify additional eligible studies. Duplicate records were removed manually. Studies were excluded if EPS parameters were incompletely reported or as broad ranges without detailed specifications. For example, studies mentioning stimulation durations such as ‘24–72 h’ without indicating the precise duration used in each experiment were excluded.

Two independent reviewers screened titles and abstracts for the use of EPS specifically in the C2C12 cell line. Extracted data included EPS parameters (voltage (V), frequency (Hz), pulse duration (ms) and total EPS time (h)), biological parameters measured (e.g., glucose uptake, AMPK phosphorylation or respiration) and used methodologies (e.g., 2‐deoxyglucose, western blot or Seahorse respirometry). All these data are summarized in Table 1.

**TABLE 1:** An overview of settings used for EPS in all included studies.

Study	EPS protocol	Biological parameter measured	Technique(s) used on C2C12 cells
Abdalkader et al. (2022)	2 ms, 1 Hz, 5 or 10 V, 3 h	Cell morphology, microfluidics and myotube differentiation	Immunocytochemistry and microscopy
Abdelmoez et al. (2020)	2 ms, 1 Hz, 40 V, 3 h	Glucose uptake, proliferation, lactate production, glycogen synthesis, fatty acid and glucose oxidation, oxygen consumption, myofibre structure, and gene expression	2‐Deoxyglucose uptake assay, BrdU assay, lactate assay, d[U‐^14^C]glucose assay, Seahorse assay, immunocytochemistry and transcriptomics
Barlow et al. (2018)	2 ms, 1 Hz, 11.5 V, 24 h	Glucose uptake, IL‐6 and insulin secretion, and cell density	2‐Deoxyglucose uptake assay, time‐resolved fluorescence IL‐6 and insulin assays and immunocytochemistry
Barlow & Solomon (2019)	2 ms, 1 Hz, 40 V, 64 min	Glucose uptake, insulin secretion, cellular respiration, oxidative phosphorylation and β‐cell function	Time‐resolved fluorescence insulin assay, Seahorse assay, and immunocytochemistry
Bayol et al. (2005)	6 ms, 2 Hz, 3 V, 48 h	Myosin heavy chain isoforms, IGF‐1 signalling and morphology	Northern blot and microscopy
Beiter et al. (2018)	2 ms, 1 Hz, 14 V, 1.5 h	Inflammation, metabolic adaptations, AMPK activity, MyHC‐IIA myosin isoform (Myh2), cell viability, glycogen content and AKT signalling	RT‐qPCR, LDH assay, oxidase‐based glycogen assay kit and ELISA
Chang & Kong (2020)	2 ms, 1 Hz, 11.5 V, 24 h or 1 ms, 99 Hz, 11.5 V, 20 pulses every 20 s, 24 h	Proteasome activity analysis, muscle atrophy, cell size, AMPK activity, irisin expression, AKT signalling, and IGF‐1 signalling	Western blot, proteasome analysis, and microscopy
Chen et al. (2019)	4 ms, 1 Hz, 20 V, 16 h	Cytokine/myokine secretion and contractile activity	Bioplex assay, movement index, RT‐qPCR and immunofluorescence
Chien et al. (2020)	2 ms, 1 Hz, 11.5 V, 16 h	Cell viability, ATP production and glucose uptake	Cell viability assay, luciferase assay, western blot and 2‐deoxyglucose uptake assay
Danilov et al. (2017)	10 ms, 1 Hz, 40 V, 4 h	Intracellular Na^+^ and K^+^ and cell viability	Western blot, atomic absorption spectrometry and cell viability assay
Ducharme et al. (2023)	2 ms, 1 Hz, 12 V, 8 h + 16 h restitution	Gene expression, myotube atrophy, cytokine/myokine secretion and TLR4, myotube contraction	RT‐qPCR, western blot, immunocytochemistry and cytokine assay
Evers‐Van Gogh et al. (2015)	2 ms, 1 Hz, 11.5 V, 24 h	AMPK activation, secretion of IL‐6, MCP‐1 and KC, and gene expression	Western blot, ELISA and RT‐qPCR
Farmawati et al. (2013)	2 ms, 0.1 or 1 Hz, 40 V, 24 h	IL‐6 secretion, glycogen content and NFAT phosphorylation	ELISA, RT‐qPCR, glycogen assay, and western blot
Fernández‐Verdejo et al. (2017)	2 ms, 1 Hz, 20 V, 4 h	Chemokine expression, ATF3 expression and ATF3, and NFkB binding site analysis	Western blot and RT‐qPCR
Fujita et al. (2007)	24 ms, 0.5 or 1 Hz, 40 V, 9 h	Ca^2+^oscillation, sarcomere assembly and contraction	Calcium imaging, movement index, western blot, and immunocytochemistry
Fujita et al. (2021)	2 ms, 1 Hz, 1 V, 4 days + reapplication 8 h after EPS termination	EPS effects on gene expression	Transcriptomics
Fukushima et al. (2021)	5 ms, (1 or 10 pulses at) 1 Hz, 20 V, 24 h	EPS and clenbuterol effects on gene expression	RT‐qPCR and transcriptomics
Gao et al. (2020)	2 ms, 1 Hz, 15 V, 1 h	Mitophagy, mitochondrial function, FUNDC1, and AMPK‐ULK1 activity	Cytotoxicity assay, immunocytochemistry, citrate synthase assay and western blot
Guo et al. (2022)	2 ms, 1 Hz, 20 V, 12 h	Cell viability and expression of VEGFB, Bcl‐2, and Bax	Western blot, RT‐qPCR, immunocytochemistry and siRNA transfection
Hashimoto et al. (2024)	2 ms, 1 Hz, 11.5 V, 24 h	Expression of VDR and regulators of cell growth and inflammation	Western blot and RT‐qPCR
Hoshino et al. (2020)	3 ms, 0, 2, or 20 Hz, 50 V, 16 h	EPS effects on metabolome and gene expression plus upstream regulators	Metabolomics, transcriptomics and western blot
Hu et al. (2018)	24 ms, 1 Hz, 20 V, 1 h	GLUT4 translocation and Rac1‐AKT signalling	Western blot and siRNA transfection
Ishiuchi et al. (2018)	2 ms, 1 Hz, 20 V, 24 h	EPS effects on myokine secretion, CXCL10 signalling and cell viability	Cytokine array, ELISA, RT‐qPCR and MTT assay,
Ishiuchi‐Sato & Nedachi (2021)	2 ms, 1 Hz, 20 V, 24 h	Effects of EPS‐stimulated C2C12 culture medium on collagen secretion and the role of CXCL10	RT‐qPCR, collagen assay and ELISA
Karvinen et al. (2022)	2 ms, 1 Hz, 12 V, 24 h	Effects of BCAAs on lipid oxidation, lipogenesis, cell viability and metabolites	Lipid oxidation assay ([9,10‐^3^H(N)] oleic acid), lipogenesis assay (^3^H‐acetate), immunocytochemistry, citrate synthase and LDH assays, and metabolomics
Kemler et al. (2020)	25 ms, 6 Hz (every 5 s), 10 V, 1 h	Circadian rhythms and clock genes	Luciferase assay, transfection and RT‐qPCR
Klymenko et al. (2020)	2 ms, 1 Hz, 11.5 V, 24 h	Effects of HDAC5 on insulin signalling, gene expression, glucose uptake, glycogen synthesis, cytokine/myokine production, AKT, and IL‐6 signalling	ELISA, western blot, RT‐qPCR, transcriptomics, 2‐deoxyglucose uptake assay, glycogen synthesis assay (^14^C‐glucose), shRNA‐transfection, luciferase assay, and ChIP analysis
Lautaoja et al. (2023)	2 ms, 1 Hz, 12 V, 24 h	Effects of glucose availability on EPS‐mediated effects on extracellular vesicles (cargo), inflammation and myokines	Transcriptomics, western blot, RT‐qPCR and extracellular vesicle isolation
Lautaoja‐Kivipelto et al. (2024)	2 ms, 1 Hz, 12 V, 24 h	PLIN5, PGC‐1𝛼, human studies and BCAA metabolism	Western blot, transcriptomics and immunocytochemistry
Lee et al. (2019)	5 ms, 1 Hz, 25 V, 2 h	Role of ATP synthase inhibitory factor 1 (IF1), extracellular ATP levels, glucose uptake, Myc‐GLUT4 translocation, Rac1 activity, intracellular calcium concentration, AMPK and AKT signalling	Western blot, siRNA transfection, RT‐qPCR, 2‐deoxyglucose uptake assay, immunocytochemistry calcium imaging and ELISA
Lee et al. (2022)	2 ms, 1 Hz, 11.5 V, 24 h	Effects of EPS on transcriptome and exercise biomarker identification	Transcriptomics, immunocytochemistry, RT‐qPCR and ELISA
Li et al. (2018)	24 ms, 1 Hz, 20 V, 1 h	Glut4 translocation, the roles of AMPK, AS160 and CaMKII, glycogen measurement, glucose uptake, lactate measurements, and nucleotide measurement	RT‐qPCR, adenoviral infection, siRNA transfection, western blot and 2‐deoxyglucose uptake assay
Li et al. (2021)	2 ms, 1 Hz, 40 V, 3 h	Effects of EPS on oleic acid and palmitic acid exposure, cell TG content and lipid metabolism	RT‐qPCR, immunocytochemistry, TG enzyme assay kit and Oil red O staining
Liu, Qi, et al. (2022)	24 ms, 1 Hz, 20 V, 1 h	Role of kalirin, Rac1 and CAMKII in contraction induced glucose uptake	siRNA transfection, western blot, RT‐qPCR and 2‐deoxyglucose uptake assay
Liu, Zhang, et al. (2022)	24 ms, 1 Hz, 20 V, 1 h	Contraction induced AKT signalling/phosphorylation, Rac1 signalling, CAMKII and glucose uptake	siRNA transfection, western blot and 2‐deoxyglucose uptake assay
Marotta et al. (2004)	30 ms, 3 Hz, 50 V, 1.5 h	Effects of EPS on enzyme activity, metabolic markers, glucose and glycogen availability	Glucose, lactate and glycogen synthesis assays
Miyatake et al. (2014)	30 ms, 1 Hz, 20 V, 1 h	Role of MIF in muscle contraction and glucose transport, cell viability and chemokine production	Plasmid transfection, western blot, LDH and 2‐deoxyglucose uptake assay
Molt et al. (2014)	10 ms, 1 Hz, 10 V, 5 h	Roles of filamin C, Xin and Aciculin in myofibril assembly, and Z‐disc structure	Western blot, immunocytochemistry, transfection, surface plasmon resonance analysis and solid‐phase protein‐binding assay
Murata et al. (2023)	2 ms, 1 Hz, 10 V, 24 h or 1 ms, 30 Hz (every 30 s), 10 V, 24 h	Effects of EPS on extracellular vesicles and calcium signalling, expression of IL‐6, Tsg101, Alix, CD63, Rab11a, Rab27a/b, Arrdc1 and Arf6	Calcium imaging, immunocytochemistry, miRNA sequencing, western blot, RT‐qPCR and extracellular vesicle isolation
Nieuwoudt et al. (2017)	6 ms, 1 Hz, 1.5 V, 16 h	Effects of contraction on palmitate induced insulin resistance, glucose uptake and PI3K–AKT signalling	Western blot, 2‐deoxyglucose uptake assay and video analysis
Pesta et al. (2021)	2 ms, 1 Hz, 11.5 V, 24 h	Insulin signalling, mitochondrial function, genetic polymorphisms (*NDUFB6*) and AKT signalling	siRNA transfection, Oroboros respirometry, RT‐qPCR and western blot
Philp et al. (2011)	0.3 ms, 1 Hz, 40 V, 3 h	Role of PGC‐1α in the effects of EPS on cellular respiration, glucose uptake, mtDNA content, mitochondrial enzyme activity, cell proliferation, morphology, palmitate uptake and oxidation	Plasmid transfection, MMT assay, western blot, RT‐qPCR, 2‐deoxyglucose uptake assay, Seahorse respiration assay and mitochondrial enzyme activity assays
Sidorenko et al. (2018)	10 ms, 1 Hz, 40 V, 2 h	Effects of EPS on Ca^2+^ oscillation and Na^+^/K^+^ ratio and downstream signalling	Transcriptomics, calcium imaging, and western blot
Son et al. (2019)	2 ms, 1 Hz, 40 V, 24 h	AR signalling, AMPK activity, testosterone production and conversion, myostatin, IL‐6, and STAT3 expression	RT‐qPCR, western blot and ELISA
Sugimoto et al. (2022)	2 ms, 66 Hz (on/off every 5s), 13 V, 3 h	Effects of EPS on myokine secretion and lactate production	Western blot and glucose and lactate assays
Tamura et al. (2020a)	25 ms, 1 Hz, 35 V, 3 h	Effects of EPS‐stimulated C2C12 culture medium on cell viability, AMPK signalling, lipolysis, adiponectin secretion, PPARγ signalling and adipogenesis	LDH assay, Oil red O staining, lipolysis assay, western blot, ELISA and RT‐qPCR,
Tamura et al. (2020b)	2 ms, 2 Hz, 13 V, 3 h or 2 ms, 66 Hz (on/off every 5s), 13 V, 3 h	Myotube contraction effects (twitch vs. tetanic) on, glycolysis, glucose oxidation capacity and mitochondrial adaptation	Western blot, glycogen/glucose/lactate assays, transcriptomics and video analysis
Thelen et al. (1997)	6 ms, 2 Hz, 3 V, 48 h	Effects of EPS on SERCA1 protein activity and expression	Transfection and ELISA
Whitham et al. (2012)	10 ms, 1 Hz, 40 V, 90 min, 4, 6, and 12 h	IL‐6 expression, secretion and IKK activity	RT‐qPCR, ELISA, western blot and ChIP assay
Wu et al. (2023)	2 ms, 1 Hz, 10 V, 2, or 24 h	Role of transthyretin in EPS induced adaptations, ATPase activity, calcium signalling, insulin sensitivity, myofibre composition, unfolded protein response and PGC1α signalling	Western blot, transfection, RT‐qPCR, immunocytochemistry and transcriptomics
Yue et al. (2020)	24 ms, 1 Hz, 20 V, 1 h	Contraction induced glucose uptake, Rac1 signalling, glucose uptake and Axin1	Western blot, siRNA transfection, Rac1 activity assay, immunoprecipitation, immunocytochemistry and 2‐deoxyglucose uptake assay
Yue et al. (2021)	24 ms, 1 Hz, 20 V, 1 h	Tiam1‐Rac1 activity, AMPK signalling, glucose uptake and GLUT4 translocation	2‐Deoxyglucose uptake assay, western blot, RT‐qPCR, transfection and Rac1 activity assay
Zhang et al. (2023)	6 ms, 1 Hz, 1.5 V, 16 h	EPS‐conditioned medium, GDF15, insulin secretion, glycolysis, ADP/ATP ratio, intracellular calcium, cell viability, cytotoxicity and cellular proliferation	Transcriptomics, ELISA, RT‐qPCR, flow cytometry, cytotoxicity assays and Seahorse respirometry assay
Zhao et al. (2018)	24 ms, 1 Hz, 20 V, 12 h	EPS‐conditioned medium, macrophage migration, cell viability, cytokine/myokine production, endothelial dysfunction, insulin resistance and apoptosis	MTS assay, macrophage migration assay, RT‐qPCR, western blot, flow cytometry and nitric oxide assay

*Note*: Parameters included are the used EPS protocol, biological parameter studied in the paper and the experimental technique used to perform the measurement.

### 2.2 Study selection

**FIGURE 1:**
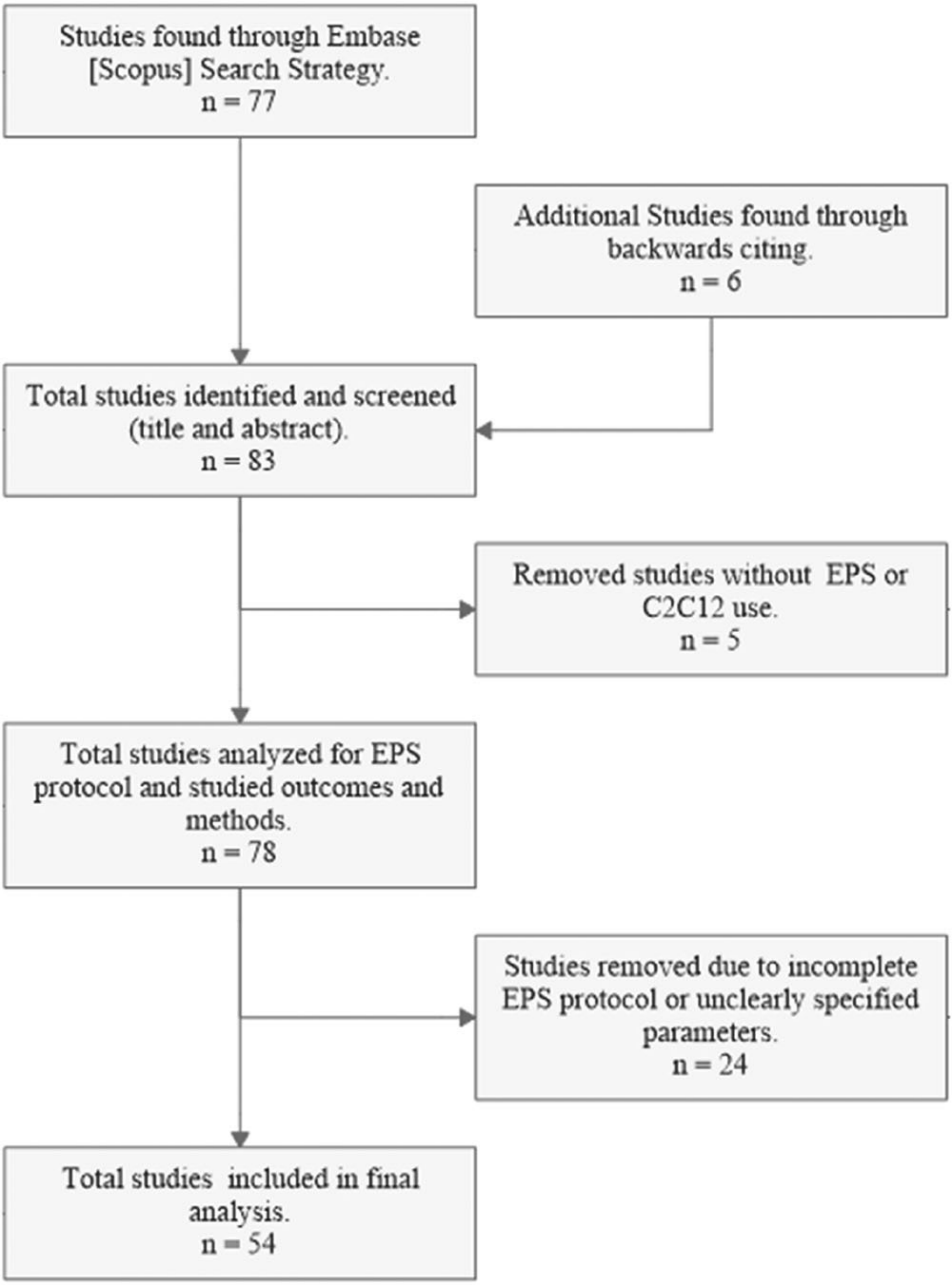
Flow diagram outlining the study selection process for inclusion in the analysis.

A total of 77 studies were initially identified through a structured search of the Embase (Scopus) database. An additional six studies were retrieved through backward citation by reviewing the reference lists of relevant articles, resulting in a total of 83 studies screened by title and abstract. Of these 83 studies, five were excluded because they did not involve EPS or the use of the C2C12 cell line, yielding 78 studies for full‐text analysis. These remaining studies were assessed for details on the EPS protocol and the reported outcomes. During full‐text review, 24 studies were excluded in accordance with the predefined exclusion criteria due to incomplete EPS protocols or vague specification of stimulation parameters. Thus, 54 studies met the inclusion criteria and were included in the final qualitative analysis (Figure 1).

### 2.3 Statistical analysis

Statistical analyses were performed using GraphPad Prism version 10 (GraphPad Software, Boston, MA, USA). Correlations between EPS parameters were calculated using a Pearson's correlation. A *P*‐value < 0.05 was considered statistically significant. Pearson's *R* values and corresponding *P*‐values are reported.

## 3 RESULTS

### 3.1 Overview of included studies, studied biological processes and employed techniques

**FIGURE 2:**
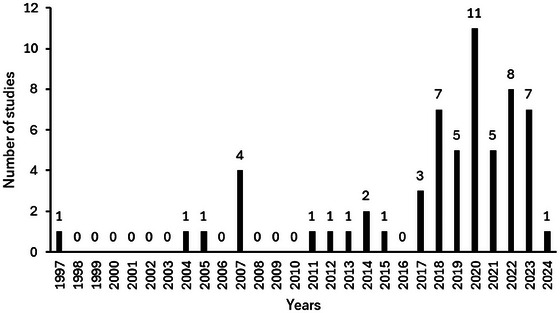
Number of peer‐reviewed publications per year.

A detailed overview of the EPS protocols used, biological parameters measured and techniques used within the 54 included studies is provided in Table 1. We observed an upwards trend for the number of publications reporting the implementation of EPS in C2C12 cells over the past three decades (Figure 2). The earliest included study was published in 1997, and from 2017 onwards the number of publications strongly increased.

**FIGURE 3:**
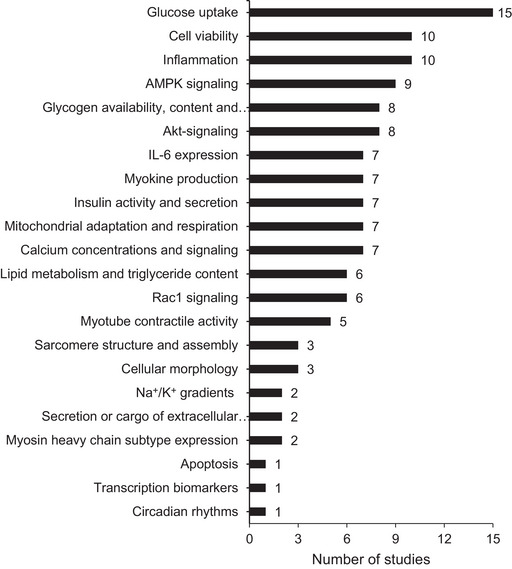
Overview of number of studies on different biological processes. Biological processes are ranked from the largest number of studies (*n* = 15) to the smallest (*n* = 1). A single paper was allowed to contribute to multiple biological categories. Details of studies per biological process are shown in Table 2.

The biological processes investigated in the included studies were characterized and the corresponding number of studies that investigated each biological process using EPS was counted (Table 2). A total of 22 different biological processes were identified, which were used for categorizing the 54 studies (Figure 3). If multiple biological processes were studied within a single study, the study was allocated to each biological process. A detailed overview of the allocation of the 54 included studies to the 22 biological processes is provided in Table 2.

**TABLE 2:** Overview of studies allocated to each of 22 identified biological parameters.

Biological process	Studies (*n*)	Studies that investigated the biological processes
Glucose uptake	15	Abdelmoez et al. (2020), Barlow et al. (2018), Barlow and Solomon (2019), Chien et al. (2020), Fujita et al. (2007), Lee et al. (2019), Klymenko et al. (2020), Li et al. (2018), Liu et al. (2022a), Liu et al. (2022b), Miyatake et al. (2014), Philp et al. (2011), Tamura et al. (2020b), Yue et al. (2020), Yue et al. (2021)
Cell viability	10	Beiter et al. (2018), Chien et al. (2020), Danilov et al. (2017), Ishiuchi et al. (2018), Karvinen et al. (2022), Miyatake et al. (2014), Sidorenko et al. (2018), Tamura et al. (2020b), Zhang et al. (2023), Zhao et al. (2018)
Inflammation	10	Beiter et al. (2018), Chen et al. (2019), Ducharme et al. (2023), Férnandez‐Verdejo et al. (2017), Ishiuchi et al. (2018), Ishiuchi et al. (2021), Klymenko et al. (2020), Lautaoja‐Kivipelto et al. (2023), Miyatake et al. (2014), Zhao et al. (2018)
AMPK activation	9	Beiter et al. (2018), Chang & Kong (2020), Evers‐van Gogh et al. (2015), Gao et al. (2020), Ishiuchi et al. (2018), Lee et al. (2019), Son et al. (2019), Tamura et al. (2020a), Yue et al. (2021)
Glycogen availability, content and synthesis	8	Abdelmoez et al. (2020), Beiter et al. (2018), Farmawati et al. (2013), Hoshino et al. (2020), Klymenko et al. (2020), Marotta et al. (2004), Tamura et al. (2020b), Zhang et al. (2023)
Akt‐signalling	8	Beiter et al. (2018), Chang & Kong (2020), Fukushima et al. (2021), Hu et al. (2018), Klymenko et al. (2020), Liu et al. (2022), Nieuwhoudt et al. (2017), Pesta et al. (2021)
IL‐6 expression	7	Barlow et al. (2018), Evers‐van Gogh et al. (2015), Farmawati et al. (2013), Hashimoto et al. (2024), Murata et al. (2023), Son et al. (2019), Whitham et al. (2012)
Myokine production	7	Chen et al. (2019), Ducharme et al. (2023), Ishiuchi et al. (2018), Klymenko et al. (2020), Lautaoja‐Kivipelto et al. (2023), Sugimoto et al. (2022), Zhao et al. (2018)
Insulin activity and secretion	7	Barlow et al. (2018), Barlow and Solomon (2019), Klymenko et al. (2020), Pesta et al. (2021), Wu et al. (2023), Zhang et al. (2023), Zhao et al. (2018)
Mitochondrial adaptation and respiration	7	Abdelmoez et al. (2020), Barlow and Solomon (2019), Beiter et al. (2018), Fukushima et al. (2021), Gao et al. (2020), Philp et al. (2011), Tamura et al. (2020b)
Calcium concentrations and signalling	7	Hoshino et al. (2020), Lee et al. (2019), Murata et al. (2023), Sidorenko et al. (2018), Thelen et al. (1997), Wu et al. (2023), Zhang et al. (2023)
Lipid metabolism and triglyceride content	6	Abdelmoez et al. (2020), Chien et al. (2020), Karvinen et al. (2022), Li et al. (2021), Philp et al. (2011), Tamura et al. (2020a)
Rac1 activity	6	Hu et al. (2018), Lee et al. (2019), Liu et al. (2022a), Liu et al. (2022b), Yue et al. (2020), Yue et al. (2021)
Myotube contractile activity	5	Chen et al. (2019), Ducharme et al. (2023), Nieuwhoudt et al. (2017), Tamura et al. (2020b), Yue et al. (2020)
Sarcomere structure and assembly	3	Fujita et al. (2007), Fujita et al. (2021), Molt et al. (2014)
Cellular morphology	3	Abdalkader et al. (2022), Bayol et al. (2005), Philp et al. (2011)
Na^+^/K^+^ gradients	2	Danilov et al. (2017), Sidorenko et al. (2018)
Secretion or cargo of extracellular vesicles	2	Lautaoja‐Kivipelto et al. (2024), Murata et al. (2023)
Myosin heavy chain subtype expression	2	Bayol et al. (2005), Beiter et al. (2018)
Apoptosis	1	Guo et al. (2022)
Transcriptomics biomarkers	1	Lee et al. (2022)
Circadian rhythms	1	Kemler et al. (2020)

*Note*: All studies from the 54 analysed studies are categorized among 22 different processes. Some studies investigated multiple processes described in this table, making the total number of studies in this table greater than 54.

**FIGURE 4:**
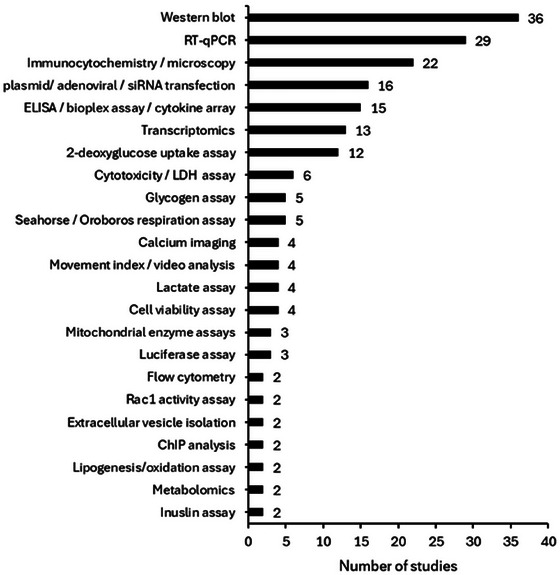
Overview of experimental techniques used in the included C2C12 cell studies. Techniques are ranked from being employed in the largest (*n* = 36) to the smallest (*n* = 2) number of studies. Techniques that were mentioned in only one study have been omitted from this figure. Details of studies per technique are shown in Table 3.

Experimental techniques employed in the included studies were extracted as well. A total of 36 different techniques were identified (Table 3). Techniques reported in more than one paper are visualized in Figure 4. Western blot was the most commonly used technique (*n* = 36 studies), followed by RT‐qPCR (*n* = 29) and immunocytochemistry (*n* = 22). Less commonly applied techniques included calcium imaging (*n* = 4), the isolation of extracellular vesicles (*n* = 2), and Rac1 activity assay (*n* = 2). A detailed overview of the specific studies employing each of the techniques is given in Table 3.

**TABLE 3:** All employed techniques were extracted from the 54 analysed studies.

Techniques	Studies (*n*)	Studies that employed the technique
Western blot	36	Chang and Kong (2020), Chien et al. (2020), Danilov et al. (2017), Ducharme et al. (2023), Evers‐Van Gogh et al. (2015), Farmawati et al. (2013), Fernández‐Verdejo et al. (2017), Fujita et al. (2007), Gao et al. (2020), Guo et al. (2022), Hashimoto et al. (2024), Hoshino et al. (2020), Hu et al. (2018), Karvinen et al. (2022), Klymenko et al. (2020), Lautaoja et al. (2023), Lee et al. (2019), Li et al. (2018), Liu et al. (2022a), Liu et al. (2022b), Miyatake et al. (2014), Molt et al. (2014), Murata et al. (2023), Nieuwoudt et al. (2017), Pesta et al. (2021), Philp et al. (2011), Sidorenko et al. (2018), Son et al. (2019), Sugimoto et al. (2022), Tamura et al. (2020a), Tamura et al. (2020b), Whitham et al. (2012), Wu et al. (2023), Yue et al. (2020), Yue et al. (2021), Zhao et al. (2018)
RT‐qPCR	29	Beiter et al. (2018), Chen et al. (2019), Ducharme et al. (2023), Evers‐Van Gogh et al. (2015), Farmawati et al. (2013), Fernández‐Verdejo et al. (2017), Fukushima et al. (2021), Guo et al. (2022), Hashimoto et al. (2024), Ishiuchi et al. (2018), Ishiuchi‐Sato and Nedachi (2021), Kemler et al. (2020), Klymenko et al. (2020), Lautaoja et al. (2023), Lee et al. (2019), Lee et al. (2022), Li et al. (2018), Li et al. (2021), Liu et al. (2022a), Murata et al. (2023), Pesta et al. (2021), Philp et al. (2011), Son et al. (2019), Tamura et al. (2020a), Whitham et al. (2012), Wu et al. (2023), Yue et al. (2021), Zhang et al. (2023), Zhao et al. (2018)
Immunocytochemistry/microscopy	22	Abdalkader et al. (2022), Abdelmoez et al. (2020), Barlow et al. (2018), Barlow & Solomon (2019), Bayol et al. (2005), Chang and Kong (2020), Chen et al. (2019), Chien et al. (2020), Ducharme et al. (2023), Fujita et al. (2007), Gao et al. (2020), Guo et al. (2022), Karvinen et al. (2022), Lautaoja‐Kivipelto et al. (2024), Lee et al. (2019), Lee et al. (2022), Li et al. (2021), Molt et al. (2014), Murata et al. (2023), Tamura et al. (2020a), Wu et al. (2023), Yue et al. (2020)
plasmid/adenoviral/siRNA transfection	16	Guo et al. (2022), Hu et al. (2018), Kemler et al. (2020), Klymenko et al. (2020), Lee et al. (2019), Li et al. (2018), Liu et al. (2022a), Liu et al. (2022b), Miyatake et al. (2014), Molt et al. (2014), Pesta et al. (2021), Philp et al. (2011), Thelen et al. (1997), Wu et al. (2023), Yue et al. (2020), Yue et al. (2021)
ELISA/bioplex assay/cytokine array	15	Beiter et al. (2018), Chen et al. (2019), Ducharme et al. (2023), Evers‐Van Gogh et al. (2015), Farmawati et al. (2013), Ishiuchi et al. (2018), Ishiuchi‐Sato and Nedachi (2021), Klymenko et al. (2020), Lee et al. (2019), Lee et al. (2022), Son et al. (2019), Tamura et al. (2020a), Thelen et al. (1997), Whitham et al. (2012), Zhang et al. (2023)
Transcriptomics	13	Abdelmoez et al. (2020), Fujita et al. (2021), Fukushima et al. (2021), Hoshino et al. (2020), Karvinen et al. (2022), Klymenko et al. (2020), Lautaoja et al. (2023), Lee et al. (2022), Murata et al. (2023), Sidorenko et al. (2018), Tamura et al. (2020b), Wu et al. (2023), Zhang et al. (2023)
2‐Deoxyglucose uptake assay	12	Abdelmoez et al. (2020), Barlow et al. (2018), Klymenko et al. (2020), Lee et al. (2019), Li et al. (2018), Liu et al. (2022a), Liu et al. (2022b), Miyatake et al. (2014), Nieuwoudt et al. (2017), Philp et al. (2011), Yue et al. (2020), Yue et al. (2021)
Cytotoxicity/LDH assay	6	Beiter et al. (2018), Gao et al. (2020), Karvinen et al. (2022), Miyatake et al. (2014), Tamura et al. (2020a), Zhang et al. (2023)
Seahorse/Oroboros respiration assay	5	Abdelmoez et al. (2020), Barlow & Solomon (2019), Pesta et al. (2021), Philp et al. (2011), Zhang et al. (2023)
Glycogen assay	5	Beiter et al. (2018), Farmawati et al. (2013), Klymenko et al. (2020), Marotta et al. (2004), Tamura et al. (2020b)
Lactate assay	4	Abdelmoez et al. (2020), Marotta et al. (2004), Sugimoto et al. (2022), Tamura et al. (2020b)
Movement index/video analysis	4	Chen et al. (2019), Fujita et al. (2007), Nieuwoudt et al. (2017), Tamura et al. (2020b)
Calcium imaging	4	Fujita et al. (2007), Lee et al. (2019), Murata et al. (2023), Sidorenko et al. (2018)
Cell viability assay	4	Chien et al. (2020), Danilov et al. (2017), Ishiuchi et al. (2018), Philp et al. (2011)
Luciferase assay	3	Chien et al. (2020), Kemler et al. (2020), Klymenko et al. (2020)
Mitochondrial enzyme assays	3	Gao et al. (2020), Karvinen et al. (2022), Philp et al. (2011)
Insulin assay	2	Barlow et al. (2018), Barlow & Solomon (2019)
Metabolomics	2	Hoshino et al. (2020), Karvinen et al. (2022)
Lipogenesis/oxidation assay	2	Karvinen et al. (2022), Tamura et al. (2020a)
ChIP analysis	2	Klymenko et al. (2020), Whitham et al. (2012)
Extracellular vesicle isolation	2	Lautaoja et al. (2023), Murata et al. (2023)
Rac1 activity assay	2	Yue et al. (2020), Yue et al. (2021)
Flow cytometry	2	Zhang et al. (2023), Zhao et al. (2018)

*Note*: An overview of the (number of) studies employing each technique is given in this table. Some studies employed multiple techniques described in this table, making the total number of studies in this table greater than 54.

### 3.2 Common and uncommon settings for EPS

**FIGURE 5:**
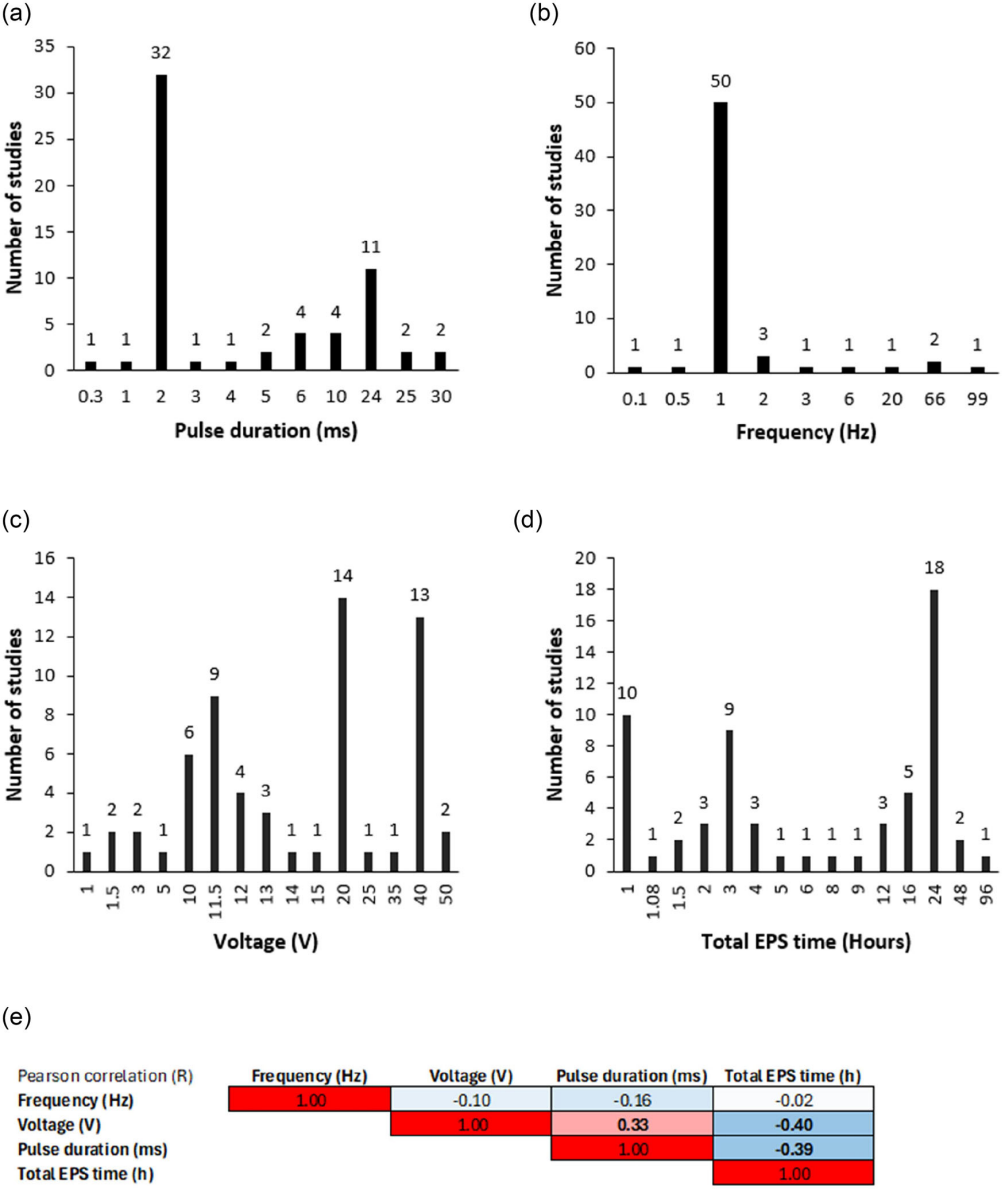
Overview of the frequency of use of specific electrical stimulation settings across all extracted protocols. (a–d) The number of protocols using specific values for (a) pulse duration, (b) frequency, (c) voltage, and (d) total electrical pulse stimulation (EPS) time. (e) Outcomes of correlation analyses between each parameter. Numbers represent Pearson correlation coefficient, with values in bold indicating statistical significance. Each data point in (a–d) reflects the number of protocols in which a specific stimulation setting was used. The total number of data points per setting represents its frequency of use across all extracted protocols. This approach accounts for studies reporting multiple protocols with varying settings.

Across all 54 analysed studies, pulse durations ranged from 0.3 to 30 ms, with the substantial majority using a pulse duration of 2 ms (*n* = 32, Figure 5a). A secondary smaller cluster was observed at a pulse duration of 24 ms (*n* = 11). The remaining 11 studies ranged between 0.3 and 30 ms, with most settings applied in only one or two studies. Stimulation frequency was 1 Hz in most studies (*n* = 50, Figure 5b). The remaining four studies used frequencies between 0.1 and 99 Hz. Less consistency in voltage settings was observed compared to frequency and pulse duration, although voltages remained within a limited span. Most studies applied 20 V (*n* = 14, Figure 5c) or 40 V (*n* = 13). Moreover, a substantial number of studies used voltages between a narrow range of 10–12 V (*n* = 19), 10 V (*n* = 6), 11.5 V (*n* = 9), 12 V (*n* = 4). Lower voltages (<10 V), including 1 V (*n* = 1), 1.5 V (*n* = 2), 3 V (*n* = 2) and 5 V (*n* = 1), were rarely used. The highest voltage used in a study was 50 V, which was applied by one study only. The total EPS time ranged from 1 to 96 h, with 24 h being most frequently used (*n* = 18, Figure 5d). Shorter protocols of 1 h (*n* = 10) or 3 h (*n* = 9) were also frequently used. Intermediate durations between 6 and 12 h were less commonly reported (*n* = 6). Finally, a few studies used multiple‐day protocols of up to 48–96 h (*n* = 3, Figure 5d).

Furthermore, Pearson correlation analysis was performed to explore potential relationships between the EPS protocol parameters (Figure 5e). A moderate negative correlation was observed between voltage and total EPS time, indicating that higher voltages generally are used in longer EPS protocols (*R* = –0.40, *P* = 0.001). A moderate positive relationship was similarly observed between voltage and pulse duration, suggesting that longer pulse bouts are associated with higher voltages (*R* = 0.33, *P* = 0.008). Conversely, a weak negative association was found between pulse duration and total EPS time, indicating that longer EPS stimulation appears to involve shorter lasting pulses (*R* = –0.39, *P* = 0.002). Finally, frequency was not significantly associated with any other parameter which remains consistent with the abovementioned finding that a 1 Hz setting is used in almost all studies (*R* = –0.16 to –0.02, all *P *> 0.05, Figure 5b).

## 4 DISCUSSION

Given the importance of skeletal muscle function in the treatment and prevention of disease, an increasing need exists to understand the molecular mechanisms at play, and the ability to investigate pharmacological and nutritional therapies that target skeletal muscle (Lautaoja et al., 2023; Walzik et al., 2024). To this end, In vitro exercise models that apply exercise‐like EPS on the C2C12 muscle cell line are increasingly utilized in research (Figure 2). Despite the translational potential of the C2C12‐EPS model, the large variety in EPS settings used throughout this research hinders comparability of results. Therefore, the current review sought to summarize the different EPS protocols described in literature, investigate which protocols are commonly used and provide an overview of the various biological processes studied by means of EPS. A total of 54 studies were selected and in‐depth analysed; they were allocated to 22 distinct studied biological processes, with glucose uptake being the most common biological process studied (Figure 3 and Table 2). Of all EPS parameters, frequency (Hz) appeared the most standardized with 50 out of 54 studies applying a frequency of 1 Hz (Figure 5b). Conversely, pulse duration (ms) was mostly divided between a lower‐end setting of 2 ms (*n* = 32) and a higher‐end setting of 24 ms (*n* = 11, Figure 5a). Similarly, the total EPS time in most experiments consisted of either shorter protocols between 1 and 3 h (*n* = 25) or longer protocols of ≥24 h (*n* = 21, Figure 5d). Finally, voltage appeared the least standardized setting as voltages ranged more widely from 1 to 50 V, with most protocols applying more moderate voltages between 10 and 20 V (Figure 5c). Moreover, Pearson correlations show some moderate positive associations between voltage and pulse duration (*R* = 0.33), while negative associations were found between voltage and total EPS time (*R* = −0.40) and pulse duration and total EPS time (*R* = −0.39, Figure 5e). Nevertheless, no associations were found between frequency and any of the other parameters (Figure 5e).

A potential explanation for the higher degree of standardization in frequency settings could lie in practical considerations. For instance, myotube contractions at a lower frequency of 1 Hz allow better visual observation compared to higher frequencies, which could produce more tetanic‐like contractions (Murata et al., 2023). Specifically, a lower stimulation frequency allows the myotubes more time for repolarization, yielding more observationally distinct contractions (Tamura et al., 2020b). However, while studying the firing frequency of neurons in human muscle, it can be observed that a contraction usually quickly increases in frequency, plateaus and then decreases over the span of several seconds (depending on the length of contraction) (De Luca & Hostage, 2010). Therefore, it remains questionable whether single 1 Hz contractions most accurately reflect in vivo muscle contraction patterns in humans and other species. Murata et al. were one of the few to apply a frequency higher than 1 Hz, and compared 1 Hz with 30 Hz contractions; the latter, included a 5 s interval for repolarization (Murata et al., 2023). Some differences were observed, such as in Ca^2+^ accumulation, but the differential responses of many biological processes to low and high frequency protocols remain unstudied (e.g., effects on insulin signalling and glucose uptake or cell growth signalling).

Despite the large variety in biological processes, some processes are more frequently studied than others. The most studied biological outcome included glucose uptake (*n* = 15, Figure 3); in addition, five studies investigated glycogen availability and metabolism without studying glucose uptake (Table 2). Thus, most studies (*n* = 20) examined glucose or glucose‐related metabolism. In addition to insulin, exercise enhances glucose uptake in skeletal muscle through mechanisms such as increased GLUT4 translocation, which was another well‐characterized measurement performed in the EPS‐C2C12 models (Stanford & Goodyear, 2014). Moreover, disturbed skeletal muscle glucose metabolism plays a central role in metabolic diseases such as type 2 diabetes and obesity, contributing to its clinical relevance (Merz & Thurmond, 2011). Yet, some topics remain under‐represented being only studied once, such as circadian rhythms. Peripheral tissues like skeletal muscle possess their own circadian clock in addition to the central circadian clock in the suprachiasmatic nucleus (Kemler, 2020). Interestingly, recent research elucidated that exercise may provide a time cue for the skeletal muscle circadian clock, possibly producing differential responses in skeletal muscle to exercise timing (Procopio & Esser, 2025). Furthermore, the secretion of extracellular vesicles is strongly influenced by exercise (Turan et al., 2023), and the use of the C2C12 model provides a valuable In vitro approach to investigate vesicle release specifically by skeletal muscle cells. Another under‐represented but physiologically important topic is muscle protein metabolism, including both protein synthesis and degradation. Surprisingly, this was not a primary focus in the analysed studies, despite its central role in muscle adaptation to exercise, ageing and disease (de Jong et al., 2023; Wall et al., 2016). A recent study (Vilchinskaya et al., 2025) highlights how EPS can modulate signalling pathways involved in muscle protein turnover, underscoring the potential of this model to explore anabolic and catabolic responses in skeletal muscle. Thus, these understudied biological processes in the C2C12‐EPS model could provide a promising avenue for future research.

The settings of voltage and pulse duration parameters varied more likely due to the diverse range of biological processes investigated in the included studies (Figure 3). These parameters directly influence strength and duration of membrane depolarization, and may hence require more careful consideration depending on the biological context being studied. For instance, depolarization‐induced calcium release necessary for muscle contractions depends largely on voltage‐gated calcium channels. Thus, insufficient voltage during stimulation may fail to produce adequate calcium ion release when studying calcium signalling (Chin, 2005). Nonetheless, some consensus exists among voltage and pulse duration settings. As mentioned above, most of the studies used voltages between 10 and 20 V, within the total observed range of 1–50 V across studies. The preference for more moderate voltages possibly occurs as extreme voltages may excessively stress cells (Bryant et al., 2016; Sun et al., 2025). The moderate negative association between voltage and total EPS time (Figure 5e) may support this idea as a longer period of stimulation in itself may provide cumulative stress, which could be balanced by lowering the voltage. Similarly, this notion was also reflected by the negative correlation between pulse duration and total EPS time. Herein, a shorter pulse duration may balance excessive electrical load and stress on cells that occur during longer EPS protocols. In contrast, a longer pulse duration during shorter EPS protocols may ensure that cells receive sufficient stimulation during the shorter time frame. Finally, the moderate positive association between voltage and pulse duration suggest that these settings benefit from co‐adjustment to enhance stimulatory effect on myotubes. Stronger stimulation could prove particularly useful in shorter EPS protocols to more closely mimic a resistance exercise regimen. On the other hand, the lower voltage and shorter pulses in long EPS protocols may resemble an aerobic endurance type exercise. However, mapping EPS protocols directly onto specific exercise modalities remains speculative, given the supra‐physiological nature of EPS (Yeo et al., 2024).

Although the C2C12 cell line is the most commonly used model for studying the effects of EPS, a limitation of this study lies in the inability to extrapolate the results to other cell lines and cell models. These include primary cells derived from human donors or mice, and the L6 rat cells (Nedachi et al., 2008). These cells likely respond differently to EPS and require other settings. Another limitation that we could not account for includes the variation in the distance between the electrodes used in the experiments. This is infrequently reported since most researchers use a six‐well plate for EPS experiments. Variation in the length of the electric field influences the electric field strength, so it is important to account for this if this deviates. However, in most cases variation in electric field length will be minimal due to the frequent use of standard six‐well plates. Strengths of this study include the large number of studies (*n* = 54) included in the analysis and the standardization of data to make comparisons across studies possible. In addition, the experimental relevance of this work lies in the detailed description of the studied biological parameters and used techniques of all included studies. Consequently, we believe this paper provides a valuable reference for future researchers using EPS in C2C12 cells.

In summary, while a frequency of 1 Hz has become largely standardized in C2C12‐EPS protocols, substantial variation was observed in voltage, pulse duration and total EPS time. The most used protocol consisted of 2 ms, 1 Hz, 11.5 V for 24 h. The predominance of glucose metabolism as the studied outcome reflects both its physiological and clinical relevance. However, other less‐studied topics such as circadian rhythms and extracellular vesicles may also provide interesting and clinically relevant avenues to pursue using the C2C12‐EPS model. Future research would benefit from clearer rationale for the employed protocol design, particularly in aligning EPS settings with cellular responses or exercise modalities. For instance, it would be interesting to further explore whether certain protocols mimic the physiological responses to resistance or endurance exercise. Taken together, this review highlights the need to interpret EPS results in context of protocol and studied outcomes, and highlight key suggestions for improving standardization, understanding parameter rationale and identifying underexplored biological applications.

## AUTHOR CONTRIBUTIONS

Conception and design: Mark R. C. van de Meene, Jelle C. B. C. de Jong, and Lars Verschuren. Acquisition, analysis and interpretation of data: All authors. Drafting and critical revision of the manuscript: All authors. All authors approved the final version. All authors confirm that they agree to be accountable for all aspects of the work, ensuring that any questions related to the accuracy or integrity of any part of the work are appropriately investigated and resolved. Furthermore, all authors confirm that they meet the criteria for authorship, and that all individuals who qualify for authorship have been included in the author list.

## CONFLICT OF INTEREST

None declared.

## FUNDING INFORMATION

No external funding was used for this work.

## Data Availability

Most data have been made available within the manuscript (Tables 1, 2, 3). Other data are available from corresponding author upon reasonable request.
